# Case Report: Intramyometrial uterine large-cell neuroendocrine carcinoma mimicking adenomyosis and leiomyomas with rapid fatal relapse

**DOI:** 10.3389/fonc.2026.1783601

**Published:** 2026-05-15

**Authors:** Xue Yi, Lirong Yuan, Shimei Sun, Ran An

**Affiliations:** 1 Department of Gynecology, Huize County People's Hospital, Qujing, Yunnan, China; 2 Department of Dermatology, The People's Hospital of Jianshui, Jianshui, Yunnan, China

**Keywords:** large-cell neuroendocrine carcinoma, uterine corpus, intramyometrial tumor, diagnostic pitfall, immunohistochemistry

## Abstract

**Background:**

Large-cell neuroendocrine carcinoma (LCNEC) of the female genital tract is rare and highly aggressive. When the dominant tumor burden is intramyometrial, preoperative imaging may mimic benign myometrial disease and delay oncologic planning.

**Case presentation:**

A 59-year-old postmenopausal woman presented with progressive lower abdominal pain for over 1 month. Laboratory testing showed elevated carcinoembryonic antigen (CEA) and cancer antigen 125 (CA125), inflammatory changes, anemia, and renal dysfunction. Preoperative transabdominal/transvaginal ultrasonography, whole-abdomen Doppler ultrasonography, chest computed tomography, and contrast-enhanced pelvic magnetic resonance imaging favored adenomyosis with multiple leiomyomas; no definite gastrointestinal or pulmonary primary lesion was identified, and no obvious metastatic lesion was detected on chest imaging. Malignant entities including uterine sarcoma, endometrial carcinoma with myometrial invasion, and metastatic disease were considered preoperatively, but the predominantly intramyometrial distribution, lack of convincing diffusion restriction, absence of overt abnormal enhancement, and nonspecific clinical findings initially argued against malignancy. The patient underwent total abdominal hysterectomy with bilateral salpingo-oophorectomy and adhesiolysis. Histopathology demonstrated high-grade LCNEC arising predominantly within the myometrium, with extrauterine spread involving parametrial tissues, the right adnexa/mesosalpinx vasculature, and tumor deposits on the bladder surface. Immunohistochemistry showed strong neuroendocrine differentiation (synaptophysin+, chromogranin A+, CD56+) with a high proliferative index (Ki-67 approximately 80%), while PAX8 and hormone receptors were negative. Adjuvant chemoradiotherapy, further staging evaluation, and subsequent molecular testing were recommended but declined. Within months, the patient developed massive pelvic–abdominal wall recurrence with fistulization and radiologic evidence of multifocal invasion, and she died approximately 7 months after surgery.

**Conclusion:**

Intramyometrial uterine LCNEC can closely resemble adenomyosis and leiomyomas on imaging and may follow an explosive clinical course. This case highlights the diagnostic pitfalls of predominantly intramyometrial disease and underscores the importance of early suspicion, multidisciplinary reassessment when clinical and imaging findings are discordant, prompt platinum-based systemic therapy when feasible, and molecular profiling if the patient is willing to proceed.

## Introduction

Neuroendocrine neoplasms (NENs) of the gynecologic tract comprise a heterogeneous and uncommon group of malignancies with a propensity for early dissemination and poor survival. Under contemporary classification frameworks, poorly differentiated neuroendocrine carcinomas (NECs) are high grade and subdivided into small-cell NEC and large-cell NEC according to cytomorphology, usually accompanied by brisk mitotic activity and a high Ki-67 index. In the uterus, LCNEC is exceptionally rare, and the published literature remains limited mainly to case reports and small series, resulting in persistent uncertainty regarding optimal staging and standardized therapy (1–3).

A particular diagnostic challenge arises when the tumor is predominantly intramyometrial with minimal or unapparent endometrial involvement. In this setting, imaging may strongly resemble adenomyosis and leiomyomas, and definitive diagnosis may only be established after hysterectomy. We report a rapidly fatal case of intramyometrial uterine LCNEC initially interpreted as benign myometrial disease on ultrasound and magnetic resonance imaging (MRI), highlighting key clinicopathologic clues, reasons for diagnostic delay, and practical management implications.

## Case presentation

**Figure 1:**
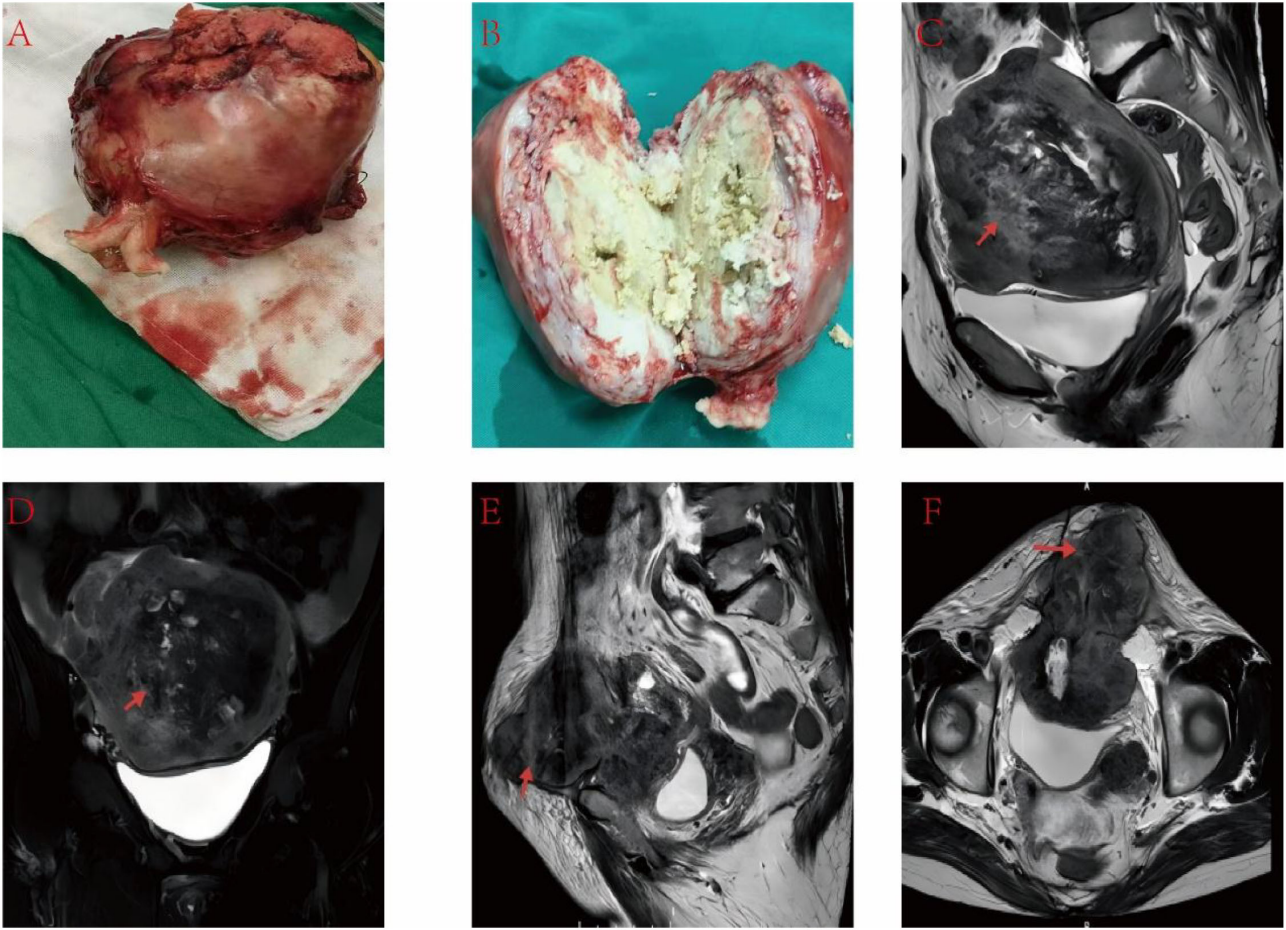
**(A)** Gross specimen after hysterectomy showing an enlarged uterus (approximately 13 × 13 × 9 cm). **(B)** Cut surface of the uterus demonstrating intramyometrial, friable, milk-white “curd-like” tumor tissue within the myometrium. **(C)** Preoperative contrast-enhanced pelvic MRI (sagittal view) showing a bulky uterine mass occupying nearly the entire uterus with loss of normal endometrial cavity configuration; the lower uterine segment/cervix, bladder, and adjacent bowel loops are compressed. **(D)** Preoperative contrast-enhanced pelvic MRI (axial view) showing a heterogeneous signal mass distorting the uterus and compressing the bladder. **(E)** Postoperative imaging showing extensive pelvic recurrence with bowel involvement and a fistulous/sinus tract communicating with the lower abdominal skin. **(F)** Postoperative contrast-enhanced pelvic MRI showing an irregular, heterogeneous recurrent mass with suspected invasion of the anterior bladder wall, associated with marked lower abdominal wall bulging.

A 59-year-old postmenopausal woman (G2P2; menopause for 16 years) presented in April 2025 with progressive lower abdominal pain for over 1 month. Her medical history was notable for chronic bronchitis and bilateral nephrolithiasis, with multiple urologic procedures for stones within the preceding year; she had undergone tubal ligation 32 years earlier. Pelvic examination revealed a firm, irregularly enlarged and tender uterus approximating a 4-month gestation size, while the adnexa were poorly appreciated. Laboratory testing showed elevated tumor markers, including CEA of 23.30 ng/mL and CA125 of 55.90 U/mL, together with inflammatory changes characterized by neutrophilia and lymphopenia, anemia, and impaired renal function with a creatinine level of 147 μmol/L and electrolyte disturbances. Transabdominal and transvaginal ultrasonography demonstrated an enlarged uterus with heterogeneous myometrial echotexture (approximately 11.3 × 11.8 × 10.1 cm), interpreted as adenomyosis with possible fibroids. Contrast-enhanced pelvic MRI similarly suggested adenomyosis with multiple leiomyomas, describing multifocal myometrial nodular lesions with mixed signal (predominantly T2 hypointense), without convincing diffusion restriction or overt abnormal enhancement; the surrounding pelvic structures were compressed and the uterine cavity was distorted (**Figures 1C–F**).

Because the CEA level was elevated, metastatic disease from an extragenital primary site, particularly from the gastrointestinal tract or lung, was considered in the preoperative differential diagnosis. The patient therefore underwent whole-abdomen Doppler ultrasonography, including gastrointestinal, urinary, and pelvic assessment, as well as chest computed tomography. These examinations did not reveal a definite gastrointestinal mass, pulmonary lesion, or other convincing primary extrauterine tumor. Uterine leiomyosarcoma and endometrial carcinoma with myometrial invasion were also considered. However, the uterine mass showed limited vascularity on ultrasound, the MRI lesions were predominantly intramyometrial and largely T2 hypointense, no obvious restricted diffusion or marked abnormal enhancement was present, chest CT did not show metastatic disease, and the patient had no abnormal uterine bleeding or vaginal discharge. Taken together, these imaging and clinical findings initially favored adenomyosis with multiple leiomyomas rather than malignancy.

Given persistent symptoms and imaging consistent with benign myometrial disease, the patient underwent total abdominal hysterectomy with bilateral salpingo-oophorectomy and adhesiolysis on May 3, 2025. Intraoperatively, no gross tumor deposits were identified on the upper abdominal viscera, and the para-aortic and pelvic lymph nodes were not palpably enlarged. The uterus was irregularly enlarged (~13 × 13 × 9 cm) with multiple whitish subserosal nodules (~2 cm) interpreted as degenerative leiomyomatous change. Dense adhesions tethered the anterior uterine wall to the anterior abdominal wall and bladder; during adhesiolysis, the adherent anterior wall tissue appeared unusually friable with “curd-like” debris. A firm right ovarian mass (~5 × 4 × 4 cm) was present and adherent to the posterior uterine wall (**Figures 1A, B**). An expanded oncologic procedure was recommended intraoperatively but was declined by the family.

**Figure 2:**
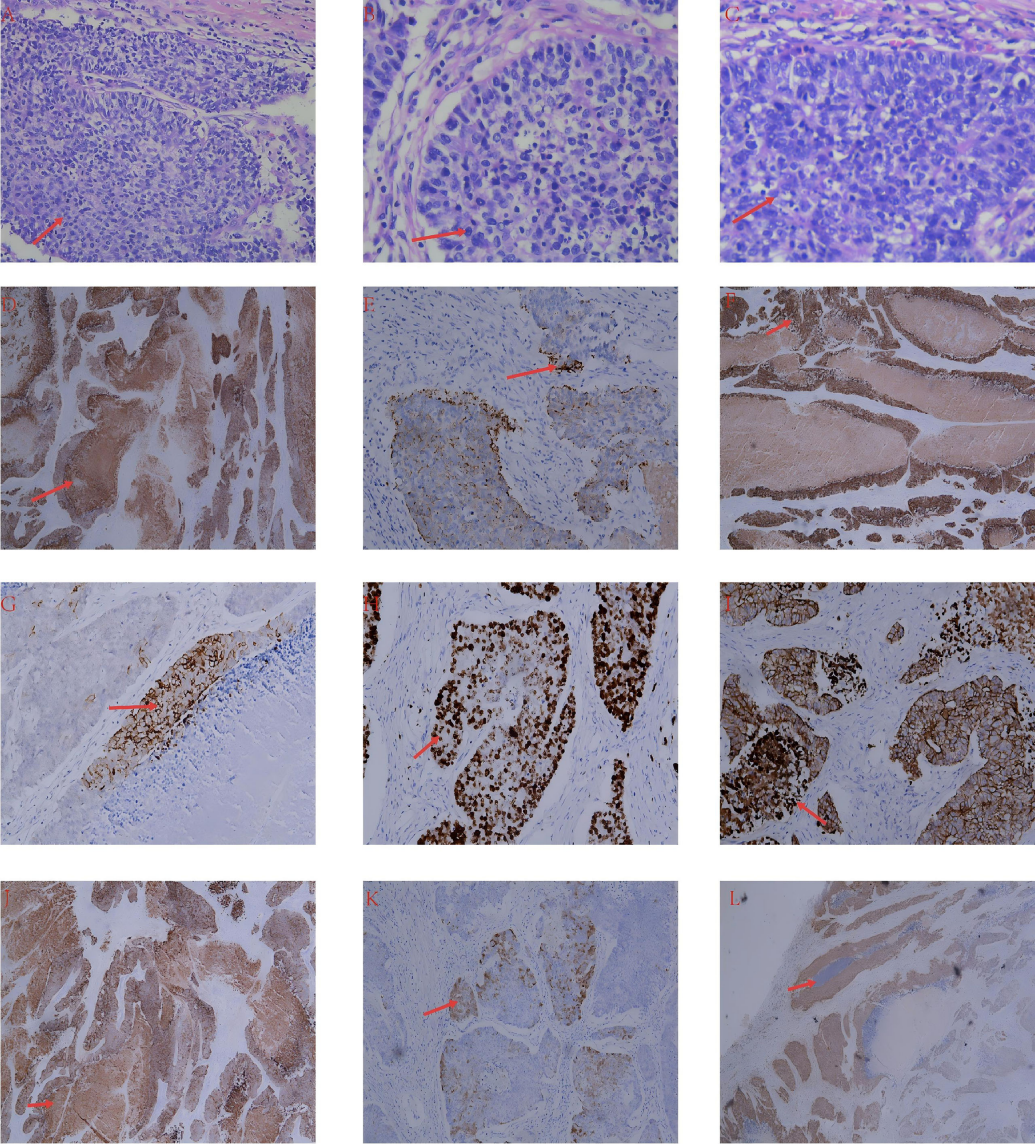
**(A–C)** Hematoxylin–eosin staining showing a high-grade malignant neoplasm composed of large atypical cells arranged in solid/nested (organoid) patterns with brisk mitotic activity and necrosis, consistent with poorly differentiated neuroendocrine carcinoma (large-cell type). **(D–L)** Tumor cells were positive for CK, L-CK, CK5/6, Syn, CgA, CD56, and p16, with Ki-67 ~80% and wild-type p53; all other tested markers were negative (vimentin, p63, WT1, GATA3, ER, PR, α-inhibin, CD30, OCT3/4, SALL4, PAX8, CK7, and TTF-1).

Histopathologic examination showed a high-grade malignant neoplasm composed of large atypical cells arranged in solid and nested organoid patterns with brisk mitotic activity and necrosis, consistent with poorly differentiated neuroendocrine carcinoma of the large-cell type. Immunohistochemistry demonstrated positivity for CK, L-CK, CK5/6, synaptophysin, chromogranin A, CD56, and p16, with Ki-67 at approximately 80% and wild-type p53. Other tested markers were negative, including vimentin, p63, WT1, GATA3, ER, PR, α-inhibin, CD30, OCT3/4, SALL4, PAX8, CK7, and TTF-1. The pathologic distribution supported an intramyometrial uterine primary with spread to parametrial tissues, the right adnexa/mesosalpinx vasculature, and tumor deposits on the bladder surface. Peritoneal washings were negative (**Figures 2A–L**). The patient’s renal dysfunction was considered multifactorial but was largely attributable to chronic bilateral stone disease with hydronephrosis and possible recurrent urinary infection. She had a history of bilateral renal calculi for more than 1 year and had undergone several prior urologic procedures, the latest occurring 11 months before this admission. Urinary ultrasonography on April 28, 2025 showed bilateral renal parenchymal echogenicity increase, left-sided moderate hydronephrosis with multiple calculi, and right-sided mild hydronephrosis with multiple calculi; right hydronephrosis possibly related to uterine compression could not be excluded. Nephrology consultation considered chronic kidney disease stage 3 and recommended renal-protective treatment, dietary adjustment, and dynamic renal monitoring. Urologic consultation further suggested recurrent stone disease with hydronephrosis and infection, right renal stones, and high surgical risk in the context of previous multidrug-resistant urinary infections. Transfer to a higher-level hospital was recommended, but the patient and her family declined. After the diagnosis was established, adjuvant chemoradiotherapy and referral for further oncologic evaluation were strongly recommended. Molecular testing was feasible through the provincial referral network and expert consultation pathway, but it was not completed. At the time of hospitalization, the patient had already stayed in hospital for 17 days, and the immunohistochemical results were still pending when she insisted on discharge despite repeated communication. After the immunohistochemical findings became available, the family was contacted by telephone, and further molecular or genomic testing was again recommended, but they declined and stated that the patient would seek care at a higher-level hospital. In October 2025, when the patient returned because of tumor recurrence, it became clear that she had remained at home and had not undergone further workup elsewhere. Transfer and additional treatment were recommended again, but the patient and her family declined further intervention.

According to the family, these decisions were strongly influenced by severe financial constraints and limited social support, as the patient came from a rural mountainous area and had no stable family income, and the couple’s only son worked away from home. The patient died approximately 7 months after surgery (**Table 1**).

**Table 1:** Timeline of clinical events.

Date	Key events
Apr 2025	Postmenopausal woman develops progressive lower abdominal pain
Apr 2025	Ultrasound/MRI interpreted as adenomyosis with multiple leiomyomas
May 2025	TAH + BSO + adhesiolysis performed
May 2025	Pathology: intramyometrial high-grade LCNEC; bladder surface and right adnexa involved; washings negative
May–Jun 2025	Adjuvant chemoradiotherapy recommended; patient declines
Sep–Oct 2025	Rapid pelvic/abdominal wall recurrence with fistulization; imaging suggests multifocal invasion/metastasis
Dec 2025	Patient dies ~7 months after surgery

## Discussion

Uterine LCNEC is exceptionally uncommon and biologically aggressive. When the dominant tumor burden is intramyometrial, as in this patient, preoperative diagnosis can be particularly difficult because both the clinical presentation and imaging findings may overlap substantially with adenomyosis and leiomyomas (1). In our case, transvaginal ultrasonography, whole-abdomen ultrasonography, contrast-enhanced pelvic MRI, and chest CT did not reveal unequivocal evidence of extrauterine primary malignancy or metastatic disease, while MRI did not show the overt diffusion restriction or conspicuous enhancement that often heighten concern for malignancy. These features contributed to an initial benign interpretation. Nevertheless, the combination of postmenopausal status, rapidly progressive pain, elevated CEA and CA125, inflammatory laboratory abnormalities, anemia, and renal dysfunction was clinically discordant and, in retrospect, should have prompted a higher index of suspicion.

The preoperative differential diagnosis included uterine leiomyosarcoma, endometrial carcinoma with myometrial invasion, and metastatic disease from a gastrointestinal or pulmonary primary. Leiomyosarcoma was considered because of the enlarged uterus and markedly heterogeneous myometrial echotexture, but the lesion was not markedly hypervascular on ultrasonography. Endometrial carcinoma could not be entirely excluded because the endometrium and uterine cavity were poorly delineated on imaging, yet the patient lacked abnormal uterine bleeding, vaginal discharge, or other typical clinical features. Metastatic disease was also considered because of the elevated CEA, but whole-abdomen ultrasonography and chest CT did not reveal a convincing gastrointestinal or pulmonary source. This pattern illustrates how predominantly intramyometrial LCNEC may evade conventional suspicion pathways and mimic common benign uterine disorders.

A definitive diagnosis of high-grade gynecologic neuroendocrine carcinoma relies on the integration of morphology and immunophenotype (1–4). LCNEC typically shows organoid nests, trabeculae, or sheets of large malignant cells with conspicuous nucleoli, extensive necrosis, brisk mitotic activity, and a high proliferative index. Immunohistochemistry usually demonstrates the expression of one or more neuroendocrine markers, most commonly synaptophysin and chromogranin A, while CD56 may provide additional support. In this patient, positivity for synaptophysin, chromogranin A, and CD56 together with a Ki-67 index of approximately 80% strongly supported a poorly differentiated, high-grade neuroendocrine carcinoma. The absence of ER, PR, and PAX8 did not exclude a uterine origin in such a poorly differentiated tumor but underscored the need for close clinicopathologic correlation and exclusion of mimics or metastases. The pathologic distribution, namely, an intramyometrial-dominant lesion with tumor deposits on the bladder surface and involvement of the adnexa and mesosalpinx vasculature, supported a uterine primary with early extrauterine dissemination (3, 4).

This case also demonstrates the practical value of paying close attention to intraoperative tissue behavior. Although imaging had favored a benign disease, the markedly friable tissue, curd-like necrotic material, and invasive adhesions encountered during surgery were not typical of ordinary leiomyoma or adenomyosis. In retrospect, these findings were highly suspicious for aggressive malignancy. When intraoperative findings are discordant with preoperative benign imaging, frozen section evaluation and immediate gynecologic oncology consultation should be considered whenever feasible.

Because prospective evidence for uterine LCNEC remains extremely limited, management is generally extrapolated from treatment paradigms for other high-grade neuroendocrine carcinomas, including cervical NEC and small-cell lung cancer (1, 3, 5). When feasible, comprehensive staging and maximal cytoreduction are usually followed by early platinum-based systemic therapy, often using a platinum–etoposide regimen, with radiotherapy considered according to disease distribution and local control requirements (6). In this patient, the pathologic findings already indicated advanced disease, and adjuvant chemoradiotherapy was strongly recommended. However, it was declined, and the subsequent explosive pelvic–abdominal wall recurrence with fistulization was consistent with the natural history of untreated high-grade NEC (7, 8).

From a practical standpoint, this case highlights several lessons. First, in postmenopausal women with rapidly progressive uterine enlargement or pain, benign-appearing imaging should be interpreted cautiously when tumor markers are abnormal or symptoms seem disproportionate. Second, a predominantly intramyometrial pattern does not exclude a highly aggressive uterine malignancy. Third, unusual intraoperative findings should trigger reconsideration of the working diagnosis and prompt oncologic escalation when possible. Finally, prompt postoperative systemic therapy and molecular profiling should be pursued whenever feasible, while recognizing that socioeconomic barriers may significantly affect adherence and access to care.

## Data Availability

The raw data supporting the conclusions of this article will be made available by the authors, without undue reservation.
